# No Effects of Cognitive Remediation on Cerebral White Matter in Individuals at Ultra-High Risk for Psychosis—A Randomized Clinical Trial

**DOI:** 10.3389/fpsyt.2020.00873

**Published:** 2020-08-28

**Authors:** Tina D. Kristensen, Bjørn H. Ebdrup, Carsten Hjorthøj, René C. W. Mandl, Jayachandra M. Raghava, Jens Richardt M. Jepsen, Birgitte Fagerlund, Louise B. Glenthøj, Christina Wenneberg, Kristine Krakauer, Christos Pantelis, Birte Y. Glenthøj, Merete Nordentoft

**Affiliations:** ^1^Copenhagen Research Center for Mental Health, CORE, Mental Health Centre Copenhagen, University of Copenhagen, Hellerup, Denmark; ^2^Center for Clinical Intervention and Neuropsychiatric Schizophrenia Research, CINS, and Center for Neuropsychiatric Schizophrenia Research, CNSR, Mental Health Centre Glostrup, University of Copenhagen, Glostrup, Denmark; ^3^Department of Clinical Medicine, Faculty of Health and Medical Sciences, University of Copenhagen, Copenhagen, Denmark; ^4^Melbourne Neuropsychiatry Center, Department of Psychiatry, The University of Melbourne, Melbourne, VIC, Australia; ^5^Section of Epidemiology, Department of Public Health, University of Copenhagen, Copenhagen, Denmark; ^6^Brain Center, University Medical Center Utrecht, Utrecht, Netherlands; ^7^Functional Imaging Unit, Department of Clinical Physiology, Nuclear Medicine and PET, University of Copenhagen, Glostrup, Denmark; ^8^Child and Adolescent Mental Health Centre, Mental Health Services, Capital Region of Denmark, University of Copenhagen, Hellerup, Denmark; ^9^Department of Psychology, Faculty of Social Sciences, University of Copenhagen, Copenhagen, Denmark

**Keywords:** ultra-high risk for psychosis, cognition, white matter, clinical trial, cognitive remediation, diffusion-weighted imaging

## Abstract

**Background:**

Individuals at ultra-high risk for psychosis (UHR) present with subtle alterations in cerebral white matter (WM), which appear to be associated with clinical and functional outcome. The effect of cognitive remediation on WM organization in UHR individuals has not been investigated previously.

**Methods:**

In a randomized, clinical trial, UHR individuals aged 18 to 40 years were assigned to treatment as usual (TAU) or TAU plus cognitive remediation for 20 weeks. Cognitive remediation comprised 20 x 2-h sessions of neurocognitive and social-cognitive training. Primary outcome was whole brain fractional anisotropy derived from diffusion weighted imaging, statistically tested as an interaction between timepoint and treatment group. Secondary outcomes were restricted to five predefined region of interest (ROI) analyses on fractional anisotropy, axial diffusivity, radial diffusivity and mean diffusivity. For significant timepoint and treatment group interactions within these five ROIs, we explored associations between longitudinal changes in WM and cognitive functions/clinical symptoms. Finally, we explored dose-response effects of cognitive remediation on WM.

**Results:**

A total of 111 UHR individuals were included. Attrition-rate was 26%. The cognitive remediation group completed on average 12 h of neurocognitive training, which was considerably lower than per protocol. We found no effect of cognitive remediation on whole-brain FA when compared to treatment as usual. Secondary ROI analyses revealed a nominal significant interaction between timepoint*treatment of AD in left medial lemniscus (P=0.016) which did not survive control for multiple comparisons. The exploratory test showed that this change in AD correlated to improvements of mental flexibility in the cognitive remediation group (p=0.001). We found no dose-response effect of neurocognitive training on WM.

**Conclusions:**

Cognitive remediation comprising 12 h of neurocognitive training on average did not improve global or regional WM organization in UHR individuals. Further investigations of duration and intensity of cognitive training as necessary prerequisites of neuroplasticity-based changes are warranted.

**Clinical Trial Registration:**

ClinicalTrials.gov, identifier NCT02098408.

## Introduction

Individuals at ultra-high risk (UHR) for psychosis present with cognitive impairments (1), intermediate between the marked cognitive deficits observed in patients with manifest psychotic disorders, and unaffected healthy controls (2–4). The cognitive impairments in UHR individuals are present across multiple domains (5), with small to large effect sizes compared to healthy controls (6).

Moreover, magnetic resonance imaging studies have revealed widespread, but subtle alterations in WM organization in UHR individuals, typically measured as lower fractional anisotropy (7). Fractional anisotropy (FA) is the currently most widely applied index of WM organization (8), sensitive to a broad range of neurobiological substrates, such as axonal density, axonal cross-section, myelin and crossing fibers (9). These WM-alterations have been associated with severity of cognitive deficits (10) and functional outcome (11), and appear to convey a liability for transition to frank psychosis (12). In a previous cross-sectional study of the current sample (10), we performed multivariate analyses of baseline data on associations between WM and cognition, comparing UHR individuals to healthy controls. At a whole-brain level, we identified an association between globally higher fractional anisotropy with a pattern of better cognitive functions in UHR individuals, but not in healthy controls. Furthermore, we identified cognitive functions associated with regional WM-interaction in five regions (fornix, medial lemniscus bilateral, left superior cerebellar peduncle, and left uncinate fasciculus) when comparing UHR individuals to healthy controls. The cognitive functions contributing reliably to the significant interactions of regional WM, were verbal intelligence, verbal fluency, planning, verbal working memory, and mental flexibility (10). The results suggested, that the underlying WM organization associated to cognitive functions were different in UHR individuals compared to healthy controls, and that specific regions were driving that difference.

Studies on patients with psychotic disorders have indicated that the relationship between WM-integrity and cognitive functions are altered by severe mental illness (13, 14). This structure-function relationship has motivated neurocognitive training programs as a means to induce neuroplastic effects on impaired cerebral networks in severe mental illness (15, 16). In a recent systematic review, we found evidence for controlled cognitive interventions, with a mean duration of 12 weeks, to be associated with WM-changes (17). One previous randomized clinical trial (RCT) on cognitive remediation in patients with schizophrenia reported regional increase in FA as an effect of treatment (18). However, there is a scarcity of studies on WM-neuroplasticity as a result of cognitive interventions in psychiatric patients, and to our knowledge none in UHR individuals. Cognitive remediation in UHR individuals is of particular interest, since these individuals may hold greater potential of brain plasticity, compared to patients with manifest psychotic disorders (19).

Cognitive remediation is a structured method of cognitive training, which is defined as “a behavioral training-based intervention that aims at improving cognitive processes (attention, memory, executive function, social cognition or metacognition) with the goal of durability and generalisation” (20). Thus, cognitive remediation targets selected cognitive domains and explicitly aims to improve social and neurocognitive functions in a manner that impacts real life functioning (21). There is meta-analytic evidence for the effectiveness of cognitive remediation in schizophrenia, with medium effect-sizes on global cognition and functional outcome (22), and the effects appear durable (20). Cognitive remediation is emphasized as an enhancing supplement to standard treatment, more effective when embedded in a broader psychiatric rehabilitation program (20). Studies are sparse in UHR-populations. A recent systematic review (23), examining six studies on cognitive remediation with UHR individuals provided some preliminary evidence for the effectiveness of cognitive remediation on cognition [e.g improvement of verbal memory (ES 0.61) (24) and processing speed (ES 0.50) (25)], and aspects of functional outcome. However, methodological considerations were raised, such as low sample sizes and high attrition rates, precluding any solid conclusions to be drawn.

In this randomized clinical trial (Function and Overall Cognition in Ultra-high risk States, the FOCUS-trial), we aimed to evaluate the effect of comprehensive neurocognitive and social cognitive remediation in UHR individuals on cerebral WM organization, as assessed by fractional anisotropy. We hypothesized, that 20 weeks of cognitive remediation in addition to treatment as usual (TAU) would increase whole-brain fractional anisotropy in UHR individuals allocated to the intervention, when compared to UHR individuals allocated to TAU. Second, we investigated the effects of cognitive remediation on FA and additional WM-measures in *a priori* ROIs. The regions were selected as the five regions our previous study had shown differentially associated to cognitive functions in UHR individuals. Next, we planned exploratory correlation-analyses between changes in WM-measures in the predefined regions, which showed significant interaction-effects on time*group and changes in clinical symptoms and cognitive functions. We expected correlations between changes in regional WM and changes in clinical symptoms/cognitive functions to be different, when comparing UHR individuals receiving cognitive remediation versus TAU. Finally, we explored a dose-response effect of cognitive remediation on WM.

## Methods

The current study is a sub study of a randomized, assessor-blinded, parallel-group, superiority clinical trial comparing TAU plus 20 weeks of intensive cognitive remediation with TAU (the FOCUS-trial, registered at ClinicalTrials.gov NCT 02098408). The main trial protocol has been published (26), and the implications of the main results have been discussed in a separate paper (27). The study was carried out at the Mental Health Centre Copenhagen and Mental Health Centre Glostrup, University of Copenhagen, Denmark. Baseline data on the relationship between cognition and WM from the FOCUS-trial have been published elsewhere (10). Here, we analyzed longitudinal data from UHR individuals at two timepoints, pre-treatment (i.e. at baseline) and post-treatment (i.e. at 26-weeks follow-up). All participants provided informed consent prior to inclusion into the study. The study protocol was approved by the Committee on Health Research Ethics of the Capital Region Denmark (study: H-6-2013-015) and the Danish Data Protection Agency (RHP-2014-009-02670).

### Procedures

Following baseline assessments, participants were randomly assigned to TAU or TAU plus cognitive remediation for 20 weeks. The randomisation was centralised and computerised with a concealed randomisation sequence carried out by the Copenhagen Trial Unit (CTU). Randomisation was stratified by current use of antipsychotic medication (yes/no) and IQ score (≤100/>100). Block size was blinded. Group allocation was concealed until the statistical analyses of the data had been completed. Investigators blinded to group allocation conducted assessments at 26-weeks (post-treatment) at a site remote from the intervention. Participants were instructed not to disclose their allocation prior to assessments. In case of failure of concealment, the assessment would be conducted by another research assessor. Assessors were psychologists and medical doctors with extensive training in the assessment instruments. Efficacy-analyses on WM were conducted by a blinded researcher.

### UHR Individuals

UHR individuals were recruited from psychiatric in-and outpatient facilities in the catchment area of Copenhagen. Individuals were help-seeking individuals aged 18 to 40 years, who fulfilled one or more of the UHR criteria as assessed by the Comprehensive Assessment of At-Risk Mental State (CAARMS) (28): attenuated psychotic symptoms, and/or brief limited intermittent psychotic symptoms, and/or trait and vulnerability state along with a significant drop or sustained low functioning for the past year. Exclusion criteria were a history with a psychotic episode of more than one week of duration; psychiatric symptoms which were explained by a physical illness with psychotropic effect or acute intoxication; a diagnosis of a serious developmental disorder, or currently receiving methylphenidate.

### Assessments

The CAARMS interview and The Structured Clinical Interview for DSM-IV Axis I Disorders (SCID-I) and part of the Structured Clinical Interview for DSM-IV Axis II Disorders (SCID-II) (29, 30) were used to diagnostically assess all UHR individuals. Level of general psychiatric symptoms was measured with the Brief Psychiatric Rating Scale Expanded Version (BPRS) (31), the level of negative symptoms was measured with the Scale for the Assessment of Negative Symptoms (SANS) (32), and the level of depressive symptoms was measured with the Montgomey-Åsberg Depression Rating Scale (MADRS) (33). Inter-rater reliability was assessed using intra-class correlations for the outcome measure SANS, BPRS, and MADRS in 12 interviews (ICC ratings from.96 to.99).

The third version of the Danish Weschler Adult Intelligence Scale (WAIS-III) (34) provided estimates of current level of intelligence. Cognitive functions were assessed using selected tests from the Brief Assessment of Cognition in Schizophrenia battery (BACS) (35): list-learning, digit sequencing, verbal fluency, and symbol coding, as well as tests from the Cambridge Neuropsychological Test Automated Battery (CANTAB) (36): Stockings of Cambridge (SOC Problems solved in minimum moves) and Intra-extradimensional set shifting test (IED Total errors adjusted, lower is better). For a detailed overview on cognitive domains and tests, see **Supplementary Table S1**.

### Image Acquisition and Processing

MRI scans were acquired on a 3 Tesla scanner (Philips Healthcare, Best, the Netherlands). Diffusion-weighted images (DWIs) were acquired using single shot spin-echo echoplanar imaging (EPI) sequence with 30 noncollinear diffusion-weighted [b = 1,000 s/mm (2)] directions and one non-diffusion weighted b=0 s/mm (2). Two DWI scans were acquired, and the latter in an opposite phase encoding direction, enabling correction for susceptibility distortions (37). Details on image acquisition and processing are provided in **Supplementary Text S2**. Tools from the FSL software library v5.0.10 (38) and MRtrix3 (www.mrtrix.org) were used for image processing. Motion parameters were extracted to correct for head motion. Maps of fractional anisotropy (FA), axial diffusivity (AD), radial diffusivity (RD) and mean diffusivity (MD) were calculated. Tract-based spatial statistics (TBSS) (39) was used to create skeleton maps using a threshold of 0.2. Using the JHU DTI-based white matter atlas labels (40), we computed the mean FA values in five WM-ROIs from skeletonized data. ROIs were selected *a priori* and constituted the five regions where we in our previous cross-sectional study of baseline data had identified aberrant associations between WM and cognitive functions: fornix, medial lemniscus bilaterally, left superior cerebellar peduncle, and left uncinate fasciculus (10). MRI quality metrics were assessed by visual inspection and calculated from each subject using a quality assessment method described in Roalf et al. (41) (**Table S3**).

### Intervention

The experimental intervention was delivered by a senior clinical psychologist with a specialization in psychotherapy and consisted of manualized neurocognitive and social cognitive remediation. The integrative approach targeting both neurocognitive and social cognitive deficits was based on the assumption, that neurocognitive and social cognitive remediation may work synergistically to increase the transfer of cognitive remediation gains to participants’ real-world functioning (42).

Duration was 2 h once a week for a total of 20 weeks in a group-setting, combined with individual home-training. In addition to the group training, the participants received 12 individual sessions designed to maximize transfer of the effect of the cognitive training to the daily lives of the participants. The approach in individual sessions were cognitive behavioral therapy and targeted everyday challenges in relation to the UHR individual’s specific cognitive deficits.

The neurocognitive remediation was performed using the Neuropsychological Educational Approach to Cognitive Remediation (NEAR) (43). Exercises from the web-based ScientificBrainTrainingpro.com and supplementary exercises from Brainhq.com were trained individually on computers for 1 h in the group-setting, followed by a group discussion aiming at relating the cognitive exercises to real world activities (bridging). UHR individuals were instructed to train the neurocognitive exercises at home to achieve the recommended dosage of 2 h per week of neurocognitive training (40 h in total). Homework was monitored in the training programs and motivation was addressed ongoing in the individual sessions, by sending text reminders and offering support from the group therapist. The therapist personalized the neurocognitive remediation based on the cognitive domains with the most severe impairments, as assessed by the UHR individuals baseline neurocognitive performance. The social cognitive training was performed using the Social Cognition and Interaction Training (SCIT) manual (44), which addresses several key social-cognitive domains. The therapist had attended a SCIT training course and received *ad hoc* supervision by Dr. David Roberts, first-author of the SCIT-manual (45). Adherence-rating to the treatment manual was conducted according to the SCIT Fidelity Scale (44) by an external rater.

Both the cognitive remediation group and the control group received TAU, which consisted of the treatment offered by the psychiatric services. TAU involved monitoring of medication, regular contact to primary practitioner delivering supportive counselling, and frequently a more regular psychotherapeutic intervention, such as social skills training, psycho-education, family-groups, supportive group-therapy, or individual therapeutic sessions with a psychologist, but not targeted cognitive remediation.

### Outcomes

The primary outcome was whole brain fractional anisotropy (FA) as an effect of interaction between timepoint and treatment-group (‘cognitive remediation versus TAU’).

Secondary outcomes were measures of white matter: FA, axial diffusivity (AD), radial diffusivity (RD), and mean diffusivity (MD) in the five ROIs fornix, medial lemniscus bilaterally, left superior cerebellar peduncle, and left uncinate fasciculus, as an effect of interaction between timepoint and treatment-group (‘cognitive remediation versus TAU’).

Planned explorative tests were correlation analyses between WM-changes in the predefined ROIs, which showed significant between-group difference on secondary outcomes, and clinical and cognitive changes as compared between treatment-groups (‘cognitive remediation versus TAU’). Moreover, as motivated by the potential dose-dependent effect of cognitive remediation on WM (17), we post-hoc tested a dose-response relationship between the proportion of cognitive remediation and change in whole-brain and regional WM.

### Statistical Analyses

The main trial planned to enroll 126 UHR individuals. Power-calculation estimating sample-size was based on cognitive outcomes for the main trial (26).

Distribution of continuous data (clinical, cognitive and demographic data) was tested for normality, and group differences were tested using general linear modelling (GLM) co-varied for age and gender. Group differences in ordinal data (tobacco smoking, alcohol, drug use, and comorbidity) were tested using the Mann–Whitney U test or Fisher’s exact test. Nominal data were tested using Pearson’s χ2 test. Group differences on baseline whole brain and regional FA were tested using univariate GLM and co-varied for age, gender, and relative and absolute head motion in the scanner.

Main analyses of treatment effect of cognitive remediation on primary and secondary outcomes were conducted using linear mixed models with repeated measurements and an unstructured covariance matrix as recommended for RCTs (46), assessing the interaction term between timepoint and group (cognitive remediation versus TAU). Covariates were age, gender, IQ, antipsychotic medication at baseline, and relative and absolute head motion in the scanner. Analyses were conducted according to the intention-to-treat principle, analyzing all participants, who completed MRI-scanning in the groups they were assigned to by randomization. Missing data were handled implicitly by the linear mixed modelling by full information maximum likelihood. Results were corrected for multiple comparisons using Bonferroni correction, and the significance threshold for the between-group analyses was set at P ≤ 0.001 (0.05/((1 whole brain + 5 regions) x 4 WM-measures x 2 treatment-groups)) (47).

Planned exploratory correlation analyses between changes in WM-measures on secondary outcomes and changes in clinical symptoms and cognitive functions, respectively, were conducted with bivariate correlation analyses using Pearson’s r or Spearmans rho as appropriate. In order to examine correlations specifically related to a potential treatment effect, testing was conducted on the predefined ROIs, which had shown an interaction-effect on time*group. Change-scores were calculated as: [numerical follow-up score – numerical baseline score]. Testing was performed with and without extreme outliers. If the main results remained unaffected, outliers were included in the analyses. Results were corrected for multiple comparisons using Bonferroni correction, and the significance threshold was set at P ≤ 0.001 (0.05/(1 ROI x 6 cognitive tests x 4 WM-measures x 2 treatment-groups)).

Exploratory post-hoc analyses to test potential dose-response effect of cognitive remediation at the follow-up were conducted with linear mixed models with repeated measurements and an unstructured covariance matrix, with covarying for number of attended neurocognitive training-hours. All analyses were covaried for age, gender, IQ, antipsychotic medication, and head motion in scanner (relative/absolute).

All analyses were performed using IBM SPSS Statistics for Windows, Version 25.0, Armonk, NY, and the significance level was set to 0.05 (two-sided).

## Results

**Figure 1:**
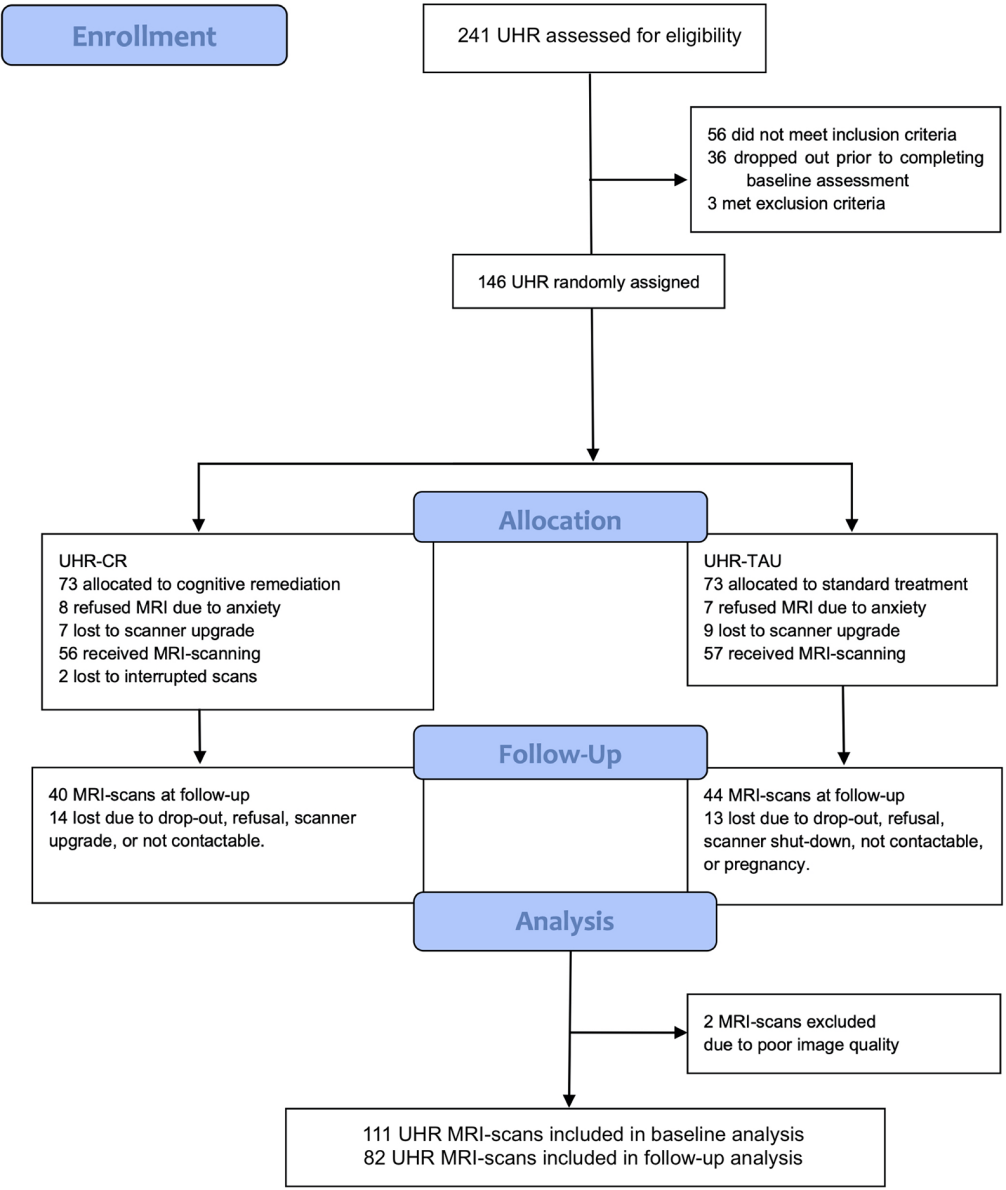
Flowchart of the FOCUS trial. CR, cognitive remediation; MRI, magnetic resonance imaging; TAU, treatment as usual; UHR, ultra-high risk.

The main trial results have been described elsewhere (no effect of cognitive remediation on primary outcome of global neurocognition, or secondary outcomes of social- and occupational functioning, negative- or depressive symptoms) (27). In short, a total of 146 UHR individuals were assigned to either TAU or TAU + cognitive remediation (see **Figure 1**). Of these, 113 were MRI-scanned, and included in the current study. Two MRI-scans were excluded due to poor image quality and extreme values. Thus, 111 UHR individuals were included in the analyses (**Figure 1**). Twenty UHR individuals discontinued the cognitive remediation intervention (18% attrition rate). Further nine UHR individuals did not complete follow-up scans due to practical issues (exceeded timeframe, scanner breakdown), resulting in a total of 82 UHR individuals completing MRI-scans at follow-up. The cognitive remediation group received significantly less TAU with a mean of 20 h within the 26 weeks intervention period compared to the TAU group (mean, 26 h). The cognitive remediation group completed a mean of 12 h of neurocognitive training. Fidelity-rating to the SCIT treatment corresponded to an excellent adherence to the SCIT therapy manual, and inter-rater reliability on the clinical outcome measures showed excellent agreement (ICC ratings from 0.96 to 0.99). No adverse events were reported relating to the intervention.

**Figure 2:**
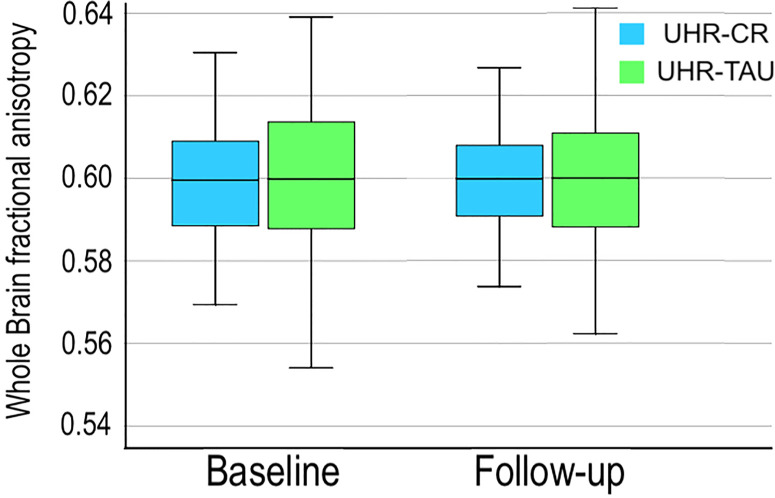
Boxplots illustrating the development of mean whole brain fractional anisotropy at baseline and 26 weeks follow-up for UHR individuals allocated to cognitive remediation versus treatment as usual are displayed. Error bars indicate the 95% confidence intervals. Note that the y-axis has been altered to enhance visual display. CR, cognitive remediation; TAU, treatment as usual; UHR, ultra-high risk.

Cognitive remediation and TAU-groups did not differ significantly on sociodemographic variables (**Table 1**), clinical symptoms or cognitive functions (**Table 2**). We found no significant difference on whole brain or regional FA between UHR individuals allocated to cognitive remediation or TAU on both timepoints (**Figure 2**). At 26-weeks follow-up, remission from UHR-status was similar in both cognitive remediation and TAU-groups (16% and 18% respectively); as well as conversion-rates to psychosis (9% and 12% respectively) (**Table 2**).

**Table 1:** Sociodemographic data at baseline for UHR individuals.

**Variable**	**UHR-CR (N = 54)**	**UHR-TAU (N = 57)**
	Mean (S.D.)/No. (Percent)	Mean (S.D.)/No. (Percent)
**Age**, mean (SD)	23.5 (4.8)	24.1 (3.6)
**Gender**		
Male	26 (48.1%)	26 (45.6%)
Female	28 (51.9%)	31 (54.4%)
**Parental SES**		
Low	6 (11.1%)	6 (10.5%)
Medium	24 (44.4%)	17 (29.8%)
High	24 (44.4%)	34 (59.6%)
**Ethnicity**		
High-Income countries	49 (92.5%)	48 (88.9%)
Low-Income countries	4 (7.4%)	6 (10.5%)
**BMI** Mean (SD)	22.6 (3.4)	24.1 (5.4)
**Handedness**		
Right	45 (83.3%)	52 (91.2%)
Left	9 (16.7%)	5 (8.8%)
**Function**		
SOFAS Mean (SD)	55.6 (11.9)	54.7 (10.1)
Activity-level (work and educational hours per week)	14.5 (17.8)	13.2 (16.2)
**Alcohol consumption (last year)**		
Daily	2 (3.7%)	1 (1.8%)
Weekly	17 (31.5%)	18 (31.6%)
Monthly	19 (35.2%)	21 (36.8%)
Once/twice	6 (11.1%)	10 (17.5%)
Never	10 (18.5%)	7 (12.3%)
**Tobacco smoking (last year)**		
Daily	25 (46.3%)	22 (38.6%)
Weekly	3 (5.6%)	3 (5.3%)
Monthly	1 (1.9%)	3 (5.3%)
Once/twice	4 (7.4%)	2 (3.5%)
Never	21 (38.9%)	27 (47.4%)
**Cannabis smoking (last year)**		
Daily	3 (5.6%)	0 (0.0%)
Weekly	1 (1.9%)	4 (7.1%)
Monthly	6 (11.1%)	1 (1.8%)
Once/twice	9 (16.7%)	8 (14.3%)
Never	35 (64.8%)	43 (76.8%)

BMI, body-mass index; CR, cognitive remediation; No., number; SD, standard deviation; SES, socio-economic status; SOFAS, social and occupational function assessment scale; TAU, treatment as usual; UHR, ultra-high risk.

**Table 2:** Clinical and cognitive data at baseline and follow-up for UHR individuals.

Variable	UHR-CR Mean (S.D.)/No. (Percent)		UHR-TAU Mean (S.D.)/No. (Percent)	
	Baseline (N = 54)	Follow-Up (N = 43)	Baseline (N = 57)	Follow-up (N = 48)
**Medication**				
Antipsychotic-naive	34 (64.2%)	16 (37.2%)	29 (50.9%)	12 (25.0%)
Current^†^ antipsychotics^★^	14 (26.4%)	24 (55.8%)	21 (36.8%)	33 (68.8%)
Current^†^ antidepressants	13 (24.5%)	12 (28.6%)	16 (28.1%)	11 (22.9%)
Current^†^ mood stabilizers	2 (3.8%)	5 (11.6%)	4 (7.0%)	4 (8.3%)
Current^†^ benzodiazepines	1 (1.9%)	6 (14.0%)	7 (12.3%)	4 (8.3%)
Diagnose of current^†^ abuse	0 (0.0%)	0 (0.0%)	1 (1.9%)	0 (0.0%)
Diagnose of current^†^ dependency	0 (0.0%)	0 (0.0%)	1 (1.9%)	0 (0.0%)
**Clinical outcomes**				
CAARMS composite	51.9 (11.9)	38.1 (16.7)	49.7 (16.5)	33.2 (15.3)
SANS	1.6 (0.7)	1.4 (0.9)	1.5 (0.8)	1.1 (1.6)
BPRS	42.9 (7.6)	37.8 (12.6)	41.0 (10.7)	38.0 (10.8)
MADRS	16.3 (7.4)	12.0 (7.5)	15.0 (7.0)	13.1 (7.2)
Remission from UHR-status		7 (16.3%)		8 (17.8%)
Conversion to psychosis		5 (9.3%)		7 (12.3%)
Drop-out		11 (20.4%)		9 (15.8%)
**Cognitive functions**				
IQ	102.8 (12.7)		104.5 (12.4)	
BACS				
List-Learning	51.7 (8.9)	53.2 (8.8)	50.4 (7.8)	53.1 (9.4)
Digit sequencing	21.0 (3.9)	21.1 (4.1)	20.0 (4.4)	21.1 (4.2)
Fluency	58.2 (15.2)	59.2 (18.6)	57.4 (11.7)	59.9 (12.3)
Symbol coding	57.3 (11.3)	62.2 (11.7)	59.0 (11.5)	62.3 (12.7)
CANTAB				
SOC	9.8 (1.7)	10.8 (2.1)	10.1 (1.8)	10.3 (1.6)
IED	18.6 (16.0)	20.1 (34.8)	19.2 (16.0)	11.4 (10.0)

The clinical characteristics and cognitive functions for UHR individuals at baseline and follow-up are shown.

^★^Atypical antipsychotics in low dose: aripiprazole, amilsulpride, olanzapine, paliperidone, quetiapine, risperidone.

† Current, the last month.

APS, attenuated psychotic symptoms; BACS, brief assessment of cognition in schizophrenia battery; BLIPS, brief limited intermittent psychotic symptoms; CAARMS, comprehensive assessment of at-risk mental state; CANTAB< Cambridge neuropsychological test automated battery; CR, cognitive remediation; IED, intra-extradimensional set shifting test, total errors, adjusted; IQ, intelligence quotient; N, count; SD, standard deviation; SOC, stockings of Cambridge, Problems solved in minimum moves; UHR, ultra-high risk.

The main mixed modelling analyses revealed no effect of the cognitive remediation on the primary outcome of whole-brain FA, with no significant interaction between timepoint*treatment (P=0.81, **Table 3**). Secondary outcome on regional WM-measures revealed a nominal significant interaction between timepoint*treatment of AD in left medial lemniscus (P=0.016, **Table 3**), which did not survive Bonferroni-correction for multiple comparisons.

**Table 3:** White matter characteristics at baseline and follow-up in individuals at ultra-high risk for psychosis.

			**FA** **Mean (SD)**		**AD** **Mean (SD)** **x10^-2^**		**RD** **Mean (SD)** **x10^-2^**		**MD** **Mean (SD)** **x10^-2^**	
			CR	TAU	CR	TAU	CR	TAU	CR	TAU
*Primary outcomes*	***Whole Brain***	**BL**	0.5782 (0.0139)	0.5805 (0.0167)	0.1360 (0.0024)	0.1361 (0.0033)	0.0462 (0.0019)	0.0459 (0.0025)	0.0761 (0.0018)	0.0759 (0.0024)
		**FU**	0.5791 (0.0160)	0.5814 (0.0160)	0.1354 (0.0021)	0.1357 (0.0028)	0.0459 (0.0022)	0.0456 (0.0023)	0.0757 (0.0019)	0.0756 (0.0021)
		Interaction Time*Group	P=0.81 F=0.06		P=0.85 F=0.04		P=0.28 F=1.18		P=0.92 F=0.01	
*Secondary outcomes*	***Fornix***	**BL**	0.5656 (0.0745)	0.5546 (0.0625)	0.1985 (0.0196)	0.2033 (0.0146)	0.0797 (0.0025)	0.0829 (0.0018)	0.1193 (0.0229)	0.1231 (0.0166)
		**FU**	0.5594 (0.0837)	0.5543 (0.0649)	0.1999 (0.0211)	0.2022 (0.0166)	0.0814 (0.0029)	0.0829 (0.0019)	0.1209 (0.0258)	0.1227 (0.0177)
		Interaction Time*Group	P=0.79 F=0.23		P=0.64 F=0.44		P=0.62 F=0.48		P=0.59 F=0.53	
	***Right medial lemniscus***	**BL**	0.6313 (0.0384)	0.6336 (0.0249)	0.1399 (0.0068)	0.1395 (0.0062)	0.0440 (0.0048)	0.0435 (0.0031)	0.0760 (0.0045)	0.0755 (0.0033)
		**FU**	0.6302 (0.0346)	0.6411 (0.0265)	0.1386 (0.0059)	0.1389 (0.0065)	0.0437 (0.0039)	0.0424 (0.0031)	0.0753 (0.0035)	0.0746 (0.0035)
		Interaction Time*Group	P=0.76 F=0.28		P=0.33 F=1.11		P=0.69 F=0.38		P=0.54 F=0.62	
	***Left medial lemniscus***	**BL**	0.6179 (0.0366)	0.6253 (0.0334)	**0.1396** **(0.0064)**	**0.1387** **(0.0067)**	0.0456 (0.0045)	0.0443 (0.0040)	0.0769 (0.0041)	0.0758 (0.0040)
		**FU**	0.6266 (0.0318)	0.6341 (0.0327)	**0.1399** **(0.0053)**	**0.1367** **(0.0072)**	0.0447 (0.0041)	0.0428 (0.0042)	0.0764 (0.0036)	0.0741 (0.0044)
		Interaction Time*Group	P=0.37 F=0.99		**P=0.02** F=3.74		P=0.41 F=0.90		P=0.14 F=2.03	
	***Left superior cerebellar peduncle***	**BL**	0.6422 (0.0319)	0.6492 (0.0373)	0.1618 (0.0071)	0.1613 (0.0074)	0.0498 (0.0046)	0.0488 (0.0059)	0.0872 (0.0043)	0.0863 (0.0054)
		**FU**	0.6403 (0.0311)	0.6472 (0.0342)	0.1602 (0.0058)	0.1593 (0.0075)	0.0495 (0.0049)	0.0486 (0.0055)	0.0864 (0.0044)	0.0855 (0.0055)
		Interaction Time*Group	P=0.40 F=0.92		P=0.18 F=1.74		P=0.17 F=1.82		P=0.12 F=2.20	
	***Left uncinate fasciculus***	**BL**	0.5034 (0.0412)	0.5027 (0.0393)	0.1277 (0.0065)	0.1279 (0.0066)	0.0541 (0.0037)	0.0543 (0.0031)	0.0786 (0.0029)	0.0788 (0.0026)
		**FU**	0.5017 (0.0431)	0.5009 (0.0369)	0.1272 (0.0054)	0.1277 (0.0067)	0.0541 (0.0042)	0.0544 (0.0029)	0.0785 (0.0030)	0.0788 (0.0026)
		Interaction Time*Group	P=0.77 F=0.27		P=0.94 F=0.06		P=0.92 F=0.08		P=0.74 F=0.31	

Mean and standard deviation (SD) of DWI data at baseline and 26-weeks follow-up for UHR individuals, comparing the cognitive remediation intervention-group (CR) to treatment as usual (TAU), and the interaction effect between timepoint*group are shown. The model comparing CR versus TAU are covaried for age, gender, IQ, antipsychotic medication, and movement in scanner. Group*Time interaction significant before correction for multiple comparisons are marked in bold.

AD, axial diffusivity; CR, cognitive remediation; DWI, diffusion weighted imaging; FA, fractional anisotropy; MD, mean diffusivity; RD, radial diffusivity; SD, standard deviation; BL, Baseline; FU, Follow-up; TAU, treatment as usual; UHR, ultra-high risk.

**Figure 3:**
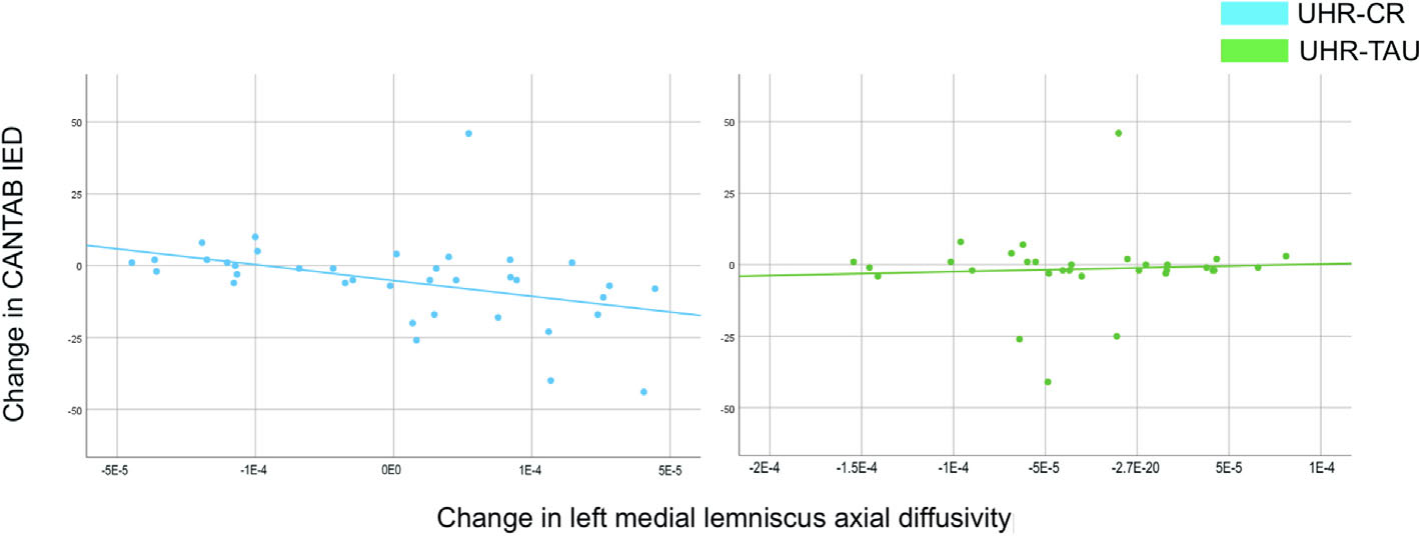
The scatterplots illustrate the exploratory correlations between changes in left medial lemniscus axial diffusivity and changes in mental flexibility (CANTAB IED total errors adjusted) in UHR individuals allocated to cognitive remediation versus treatment as usual are displayed. Error-bars indicate the 95% confidence intervals. Note that the y-axis has been altered to enhance visual display. The correlations have been tested with and without outliers, which were included due to no effect on the result. Numerical results are reported in detail in **Supplementary Table S4**. CANTAB, Cambridge neuropsychological test automated battery; CR, cognitive remediation; IED, intra-extra dimensional set-shifting; TAU, treatment as usual; UHR, ultra-high risk.

Exploratory analyses of associations between change in WM-measures in the region with significant between-group differences on secondary outcomes, and change in cognitive functions revealed a significant negative correlation between WM (AD increase) in left medial lemniscus, and changes in CANTAB IED (total errors adjusted decreased) (P=0.001, CC −0.54) for the cognitive remediation group, but not for TAU (**Figure 3**). No other correlations survived Bonferroni correction (See **Supplementary Tables S4** and **S5** for details).

In the exploratory analyses of dose-response effects in the cognitive remediation group, we found no effect when adding number of neurocognitive training hours as a covariate to WM-changes.

## Discussion

To our knowledge, this is the first randomized, clinical trial to evaluate the effect of cognitive remediation on white matter in UHR individuals. Contrary to our main hypothesis, we found no effect of cognitive remediation on the primary outcome of whole-brain FA. On our secondary outcome a nominal significant effect on one predefined white matter region (left medial lemniscus) did not survive adjustment for multiple comparisons. The lack of global and regional WM-changes as a result of cognitive remediation we found are in line with the results from the main trial (27), which did not result in improvements in global measures of cognition, function, or clinical symptoms, but marginal effects on exploratory outcomes (i.e. emotion recognition). Nor did our dose-response analyses on completed hours of neurocognitive training in the cognitive remediation group support an effect on FA in whole brain or ROIs.

The result could, beyond the conclusion of no effect of cognitive remediation on WM, be explained by the low number of neurocognitive training sessions. The average of 12 h of neurocognitive training is considerably lower than the recommended dosage (25–30 h) assumed necessary for driving cognitive improvements (48). A controlled, randomized trial on patients with schizophrenia reported the neuroplastic effects of cognitive remediation (increased FA in corpus callosum), which was correlated to cognitive executive functions (18). However, this intervention group was exposed to 40 1-h sessions of neurocognitive training with a higher frequency, which is a considerably larger dose than the UHR individuals in the current trial. The question of sufficient dosage to induce consistent WM-changes might be particularly important when evaluating neuroplasticity-based treatment. A multistage model for associations between learning and cerebral changes would explain how rapid improvements in learning and behavior may be linked to transient and elastic WM-changes (49), which tend to return to baseline at subsequent follow-ups (50). Consolidation and automatization of training and learning, which would be accompanied by more robust neuroplastic reorganization, would appear within a longer time-frame (51). Indeed, several studies have demonstrated a sustained effect on WM-measures, suggesting a temporal dynamic in progressive WM-reorganization associated with increased skills and new learning (52–54). However, the underlying biological mechanisms involved in these time-dependent variations are not straightforward, as multiple cellular processes may be associated with WM-measures such as FA. Various candidate mechanisms of activity-dependent WM-reorganization has been suggested, such as long-term potentiation (55), growth of astrocytes and oligodendrocyte precursor cells (56), myelination (57), and reorganization of axons and fiber-bundles (58). Our results stress the perspective of investigating duration and intensity as prerequisites of neuroplasticity-based interventions embedded in a broader clinical setting. Moreover, future research specifying the potential active treatment ingredients beyond duration and intensity, such as choice of remediation strategies (i.e. integrative versus simple programs, or compensatory versus restorative remediation approaches) may support the clinical tailoring of treatment strategies. The clinical perspectives includes enhancement of motivation and attendance (21, 59), as well as the question of whether this comprehensive treatment may only be viable for the subgroup of UHR individuals who can be engaged sufficiently to practice the skills in the recommended dosage.

Exploratory analyses revealed mental flexibility to be correlated with a change of left medial lemniscus AD in the cognitive remediation group, but not in the TAU-group. Although our observation is marginal and should be interpreted very cautiously, medial lemniscus is part of the cerebello-thalamocortical connections [CTC (60)], which previously has been associated with symptom course in UHR individuals (61, 62). Interestingly, our observation would be consistent with a recent study in patients with schizophrenia, reporting that WM organization in medial lemniscus predicted training induced improvements in executive functioning, and suggested that preserved WM organization in CTC-connections may be an important determinant for training induced neurocognitive plasticity (63). We speculate whether this correlation may indicate a subtle protective effect of cognitive remediation on loss of WM-integrity. The protective approach of intact cognitive functions have been discussed as an alternative to focusing on restoration of impaired functions by cognitive remediation (64). Our observations lend support to further investigations of strategies for cognitive remediation, i.e. specifically aimed at preserving targeted cognitive functions while potentially preventing WM-deterioration in early psychosis, versus the integrative program combining various remediation strategies as applied in the current trial.

A strength of this study is that it was undertaken as part of a high quality randomized, assessor-blinded clinical trial. Limiting the study is the moderate attrition-rate, along with the low number of mean neurocognitive training hours, which impede reaching firm conclusions about the effect of cognitive remediation delivered per the trial-protocol. Furthermore, sample-size was calculated on different outcomes from the main trial, which is a weakness in our study-design. However, this study presents a large sample-size compared to previous studies examining neuroplastic effects of cognitive interventions.

## Conclusion

Cognitive remediation comprising 12 h of neurocognitive training on average did not improve global or regional WM organization in UHR individuals. Further investigations of the duration and intensity of neuroplasticity-based cognitive training as necessary prerequisites of WM-changes are warranted.

## Data Availability Statement

The data sets presented in this article are not readily available because: Not allowed by the Danish Data Protection Agency. Requests to access the datasets should be directed to TK, tina.dam.kristensen@regionh.dk.

## Ethics Statement

The studies involving human participants were reviewed and approved by The study protocol was approved by the Committee on Health Research Ethics of the Capital Region Denmark (study: H-6-2013-015) and the Danish Data Protection Agency (RHP-2014-009-02670). The patients/participants provided their written informed consent to participate in this study.

## Author Contributions

MN, LG, and BF conceived and designed the trial protocol. MN, BG, LG, CH, TK, and CW raised the funding. JJ and BF supervised the data collection. BE, RM, and CP supervised the study. JR processed MRI-data. TK conducted the analyses supervised by CH. TK wrote the initial draft. All authors contributed to the article and approved the submitted version.

## Funding

The study was funded by The Danish Council for Independent Research (DFF-4004-00314); TrygFoundation (108119); the Mental Health Services in the Capital Region of Denmark; the research fund of the Capital Region of Denmark; the Lundbeck Foundation Center for Clinical Intervention and Neuropsychiatric Schizophrenia Research, CINS (R155-2013-16337). CP was supported by a NHMRC Senior Principal Research Fellowship (1105825) and by a grant from Lundbeck Foundation (R246-2016-3237).

## Conflict of Interest

BE is part of the Advisory Board of Eli Lilly Denmark A/S, Janssen-Cilag, and Takeda Pharmaceutical Company Ltd; and has received lecture fees from Bristol-Myers Squibb, Otsuka Pharma Scandinavia AB, Eli Lilly Company, and Lundbeck Pharma A/S. BG is the leader of a Lundbeck Foundation Centre of Excellence for Clinical Intervention and Neuropsychiatric Schizophrenia Research (CINS), which is partially financed by an independent grant from the Lundbeck Foundation based on international review and partially financed by the Mental Health Services in the Capital Region of Denmark, the University of Copenhagen, and other foundations. Her group has also received a research grant from Lundbeck A/S for another independent investigator-initiated study. All grants are the property of the Mental Health Services in the Capital Region of Denmark and administrated by them. She has no other conflicts to disclose.

The remaining authors declare that the research was conducted in the absence of any commercial or financial relationships that could be construed as a potential conflict of interest.
