# Supplementary material for: No Effects of Cognitive Remediation on Cerebral White Matter in Individuals at Ultra-High Risk for Psychosis—A Randomized Clinical Trial
Source: Front Psychiatry. 2020 Aug 28;11:873. doi: 10.3389/fpsyt.2020.00873 (PMC7485415; doi:10.3389/fpsyt.2020.00873)
Supplement: Supplementary file 1 [file DataSheet_1.docx]

**Supplementary tables and figures**

**Table S1: Cognitive functions by outcome measures**

| **Cognitive function** | **Test** | **Outcome measure** |
| --- | --- | --- |
| Current Verbal IQ | (WAIS-III)  Similarities subtest of Wechsler Adult Intelligence Scale | - Total score, i.e. defining a conceptual similarity between two words. |
| Verbal learning and memory | BACS  List learning | - Total number of words correctly recalled across 5 trials. |
| Verbal working memory | BACS  Digit sequencing | - Number of correctly ordered sequences of digits. |
| Verbal fluency | BACS  Verbal fluency, semantic and phonetic | - Number of correct words generated (supermarket items, F- and S-words). |
| Processing speed | BACS  Symbol coding | - Number of written symbols correctly matched with numbers. |
| Planning | CANTAB (SOC)  Stockings of Cambridge | - Problems solved in minimum moves. |
| Set shifting/mental flexibility | CANTAB (IED)  Intra-Extra-Dimensional Set-shifting | - Total errors, adjusted for the number of stages not completed. Lower is better. |

*Table S1 shows the categories of cognitive functions associated with the specific tests and outcome measures applied in the analyses.*

**Text S2 Image acquisition and processing**

Magnetic resonance imaging (MRI) scans were acquired on a 3 tesla scanner (Philips Healthcare, Best, The Netherlands), using a 32-channel SENSE head coil (Invivo, Orlando, Florida, USA). Diffusion-weighted images (DWI) were acquired using single shot spin-echo echoplanar imaging (EPI) sequence with 30 non-collinear diffusion weighted (b = 1000 s/mm^2^) scans and 5 diffusion unweighted (b = 0 s/mm^2^) scans. Other acquisition settings were: Acquisition matrix = 128 × 99 × 75; voxel dimensions = 1.88 × 2.41 × 2mm^3^, no slice gap=0; reconstructed voxel dimensions = 1.88 x 1.88 x 2mm^3;^ TR=7058 ms; TE = 68 ms; parallel imaging SENSE factor = 3(AP); flip angle = 90°. Two DWI scans were acquired, where an opposite phase encoding direction were used in the second scan, enabling correction for susceptibility distortions^2,3^. Tools from the FSL software library v5.0.10^4^ and MRtrix3 ([www.mrtrix.org](http://www.mrtrix.org)) were used for image processing. Non-brain tissue was removed using *dwi2mask*^5^. DWI data were denoised by exploiting data-redundancy in the principal component analyses (PCA) domain^6,7^. Next, we performed B1 field inhomogeneity correction^8,9^. An eddy current and susceptibility artifact correction was performed using *topup*^10^ and *eddy*^10^. We extracted absolute and relative head motion parameters from *eddy_restricted_movement_rms* output of *eddy*. We used the motion parameters to correct for head motion in the statistical analyses. Diffusion (kurtosis) tensor were computed using rotated diffusion vectors and iterative reweighted linear least squares estimator^11^. Diffusion parameter maps of FA were calculated using *tensor2metric*^12^. Tract-based spatial statistics (TBSS)^13,8^ was used to align FA data using the nonlinear image registration tool (FNIRT)^14,15^. The resulting mean FA image (threshold of 0.2) was thinned to create a mean study-specific FA skeleton template. Next, the nearest maximum FA values of all subjects were projected onto the mean study-specific FA skeleton template^13^. Subsequently, the AD, RD and MD images were non-linearly projected on the FA skeleton. Using the JHU DTI-based white-matter atlas labels^16,17^**,** we extracted the mean FA, AD, RD and MD values in 48 WM label ROIs from skeletonized data. MRI quality metrics were assessed using a quality assessment method described in Roalf et al. 2016 (Table S2).

**Table S3 Image quality metrics**

|  | **TSNR**  **Mean (SD)** | **MAXVOX**  **Mean (SD)** | **MEANVOX**  **Mean (SD)** |
| --- | --- | --- | --- |
| UHR-individuals and HCs in the full sample (N=165) | 6.98  (0.36) | 4539.6  (6203) | 863.8  (431.3) |
| Roalf et al. (N=147)  “Poor” | 5.52  (0.93) | 14497  (8667) | 2001.50  (1080.20) |
| Roalf et al. (N=468)  “Good” | 6.9  (0.68) | 7165  (7189) | 830.40  (597.10) |
| Roalf et al. (N=742)  “Excellent” | 7.37  (0.55) | 1684  (1741) | 378  (164.10) |

*Table S3 shows mean (SD) of quality metrics on DWI data for the UHR-individuals and healthy controls in the full sample, as well as for the quality assessment groups defined by Roalf et al.* ^1^*. Quality control was done by visually inspecting all DW images slice by slice before processing, and excluded if the image quality was judged to be of poor quality. Three image quality metrics (temporal signal-to-noise ratio (TSNR), maximum voxel intensity outlier count (MAXVOX) and mean voxel intensity outlier count (MEANVOX)) were calculated from each subjects DW image* *using a quality assessment method described in Roalf et al. The measured quality metrics in this study ranged between the ‘good’ and ‘excellent’ quality.*

*Abbreviations: DWI: diffusion weighted imaging; FA: fractional anisotropy; HC: healthy controls; SD: standard deviation; UHR: ultra-high risk*

**Table S4 Correlations between changes in left medial lemniscus white matter and changes in cognitive functions**

**UHR-CR testing with outliers:**

| **CC + Sign (2-tailed)** | **FA ML_L** | **AD ML_L** | **RD ML_L** | **MD ML_L** |
| --- | --- | --- | --- | --- |
| **BACS** |  |  |  |  |
| List-learning | -0.126  0.444 | 0.111  0.502 | 0.066  0.690 | 0.101  0.542 |
| Digit sequencing | 0.130  0.431 | -0.002  0.990 | -0.131  0.427 | -0.056  0.734 |
| Fluency | -0.166  0.311 | 0.256  0.116 | 0.259  0.112 | 0.302  0.062 |
| Symbol coding | -0.099  0.556 | -0.287  0.081 | -0.078  0.641 | -0.229  0.167 |
| **CANTAB** |  |  |  |  |
| SOC | -0.035  0.837 | 0.078  0.646 | 0.035  0.836 | 0.075  0.661 |
| IED | 0.110  0.519 | -0.540 **  0.001 | -0.314  0.058 | -0.470**  0.003 |

**UHR-CR testing without outliers:**

| **CC + Sign (2-tailed)** | **FA ML_L** | **AD ML_L** | **RD ML_L** | **MD ML_L** |
| --- | --- | --- | --- | --- |
| **BACS** |  |  |  |  |
| List-learning | -0.111  0.506 | 0.095  0.572 | 0.061  0.718 | 0.089  0.597 |
| Digit sequencing | 0.128  0.444 | 0.002  0.991 | -0.121  0.468 | -0.055  0.742 |
| Fluency | -0.171  0.303 | 0.239  0.148 | 0.258  0.117 | 0.296  0.071 |
| Symbol coding | -0.104  0.539 | -0.289  0.083 | -0.074  0.665 | -0.227  0.176 |
| **CANTAB** |  |  |  |  |
| SOC | -0.024  0.887 | 0.052  0.762 | 0.021  0.902 | 0.062  0.719 |
| IED | 0.102  0.556 | -0.533 **  0.001 | -0.312  0.064 | -0.467**  0.004 |

**UHR-TAU**

| **CC + Sign (2-tailed)** | **FA ML_L** | **AD ML_L** | **RD ML_L** | **MD ML_L** |
| --- | --- | --- | --- | --- |
| **BACS** |  |  |  |  |
| List-learning | -0.159  0.342 | 0.280  0.088 | 0.167  0.316 | 0.225  0.174 |
| Digit sequencing | -0.055  0.742 | -0.150  0.370 | -0.079  0.636 | -0.127  0.446 |
| Fluency | -0.046  0.785 | 0.041  0.806 | 0.010  0.954 | 0.003  0.984 |
| Symbol coding | -0.164  0.324 | -0.135  0.421 | 0.088  0.600 | -0.031  0.852 |
| **CANTAB** |  |  |  |  |
| SOC | -0.085  0.620 | 0.140  0.414 | 0.158  0.358 | 0.169  0.325 |
| IED | 0.009  0.961 | -0.021  0.909 | -0.011  0.955 | -0.030  0.872 |

*Table S4 displays the results from the bivariate correlation analyses (2-tailed significance P and correlation coefficient CC) between changes from baseline to follow-up in the white matter metrics in left medial lemniscus and changes in cognitive functions. The correlation analyses were done with and without outliers, and as the result was unaffected, one outlier was included in the analyses.*

*Abbreviations: AD: axial diffusivity; BACS: brief assessment of cognition in schizophrenia battery ; CANTAB: Cambridge neuropsychological test automated battery ; CC: correlation coefficient; CR: cognitive remediation; FA: fractional anisotropy; IED: intra-extradimensional set shifting test, Total errors, adjusted ; L: left; MD: median diffusivity; ML: medial lemniscus; RD: radial diffusivity; SD: standard deviation; SOC: stockings of Cambridge, Problems solved in minimum moves; TAU: treatment as usual.*

** significant before Bonferroni-correction (P<0.05)*

**Table S5 Correlations between changes in white matter and changes in cognitive functions**

| UHR-CR | FA ML_L  P (CC) | AD ML_L  P (CC) | RD ML_L  P (CC) | MD ML_L  P (CC) |
| --- | --- | --- | --- | --- |
| SANS total | NS | **0.028***  (0.348) | NS | 0,077  (0,283) |
| BPRS total | NS | NS | NS | NS |
| MADRS total | NS | NS | NS | NS |
| CAARMS composite | NS | NS | NS | NS |
| Remission status | NS | 0.081  (-0.283) | NS | NS |
| UHR-TAU | **FA ML_L**  **P (CC)** | **AD ML_L**  **P (CC)** | **RD ML_L**  **P (CC)** | **MD ML_L**  **P (CC)** |
| SANS total | NS | NS | NS | NS |
| BPRS total | NS | 0.088  (0.280) | NS | NS |
| MADRS total | NS | NS | NS | NS |
| CAARMS composite | NS | NS | NS | NS |
| Remission status | NS | NS | NS | NS |

*Table S5 displays the results from the bivariate correlation analyses (2-tailed significance P and correlation coefficient CC) between changes from baseline to follow-up in the white matter metrics in left medial lemniscus and changes in clinical outcomes. The correlation analyses were done with and without outliers, and as the result was unaffected, one outlier was included in the analyses.*

*Abbreviations: AD: axial diffusivity; BPRS: Brief Psychiatric Rating Scale Expanded Version; CAARMS: comprehensive assessment of at-risk mental state; CC: correlation coefficient; CR: cognitive remediation; FA: fractional anisotropy; L: left; MADRS: Montgomey-Åsberg Depression Rating Scale; MD: median diffusivity; ML: medial lemniscus; NS: non-significant; RD: radial diffusivity; SANS; Scale for the Assessment of Negative Symptoms; SD: standard deviation; TAU: treatment as usual.*

** significant before Bonferroni-correction (P<0.05)*

**References**

1 Roalf DR, Quarmley M, Elliott MA, *et al.* The Impact of Quality Assurance Assessment on Diffusion Tensor Imaging Outcomes in a Large-Scale Population-Based Cohort. *Neuroimage* 2016; **125**: 903–19.

2 Andersson JLR, Skare S, Ashburner J. How to correct susceptibility distortions in spin-echo echo-planar images: Application to diffusion tensor imaging. *Neuroimage* 2003; **20**: 870–88.

3 Skare S, Andersson JL. On the effects of gating in diffusion imaging of the brain using single shot EPI. *Magn Reson Imaging* 2001; **19**: 1125–8.

4 Jenkinson M, Beckmann CF, Behrens TEJ, Woolrich MW, Smith SM. Fsl. *Neuroimage* 2012; **62**: 782–90.

5 Dhollander T, Raffelt D, Connelly A. Unsupervised 3-tissue response function estimation from single-shell or multi-shell diffusion MR data without a co-registered T1 image. *ISMRM Work Break Barriers Diffus MRI* 2016; : 5.

6 Veraart J, Novikov DS, Christiaens D, Ades-aron B, Sijbers J, Fieremans E. Denoising of diffusion MRI using random matrix theory. *Neuroimage* 2016; **142**: 394–406.

7 Veraart J, Fieremans E, Novikov DS. Diffusion MRI noise mapping using random matrix theory. *Magn Reson Med* 2016; **76**: 1–12.

8 Smith SM, Jenkinson M, Woolrich MW, *et al.* Advances in functional and structural MR image analysis and implementation as FSL. *Neuroimage* 2004; **23(S1)**: 208–19.

9 Zhang Y, Brady M, Smith S. Segmentation of brain MR images through a hidden Markov random field model and the expectation-maximization algorithm. *IEEE TransMedImaging* 2001; **20**: 45–57.

10 Andersson JLR, Sotiropoulos SN. An integrated approach to correction for off-resonance effects and subject movement in diffusion MR imaging. *Neuroimage* 2016; **125**: 1063–78.

11 Veraart J, Sijbers J, Sunaert S, Leemans A, Jeurissen B. Weighted linear least squares estimation of diffusion MRI parameters: Strengths, limitations, and pitfalls. *Neuroimage* 2013; **81**: 335–46.

12 Basser PJ, Mattiello J, LeBihan D. MR diffusion tensor spectroscopy and imaging. *Biophys J* 1994; **66**: 259–67.

13 Smith SM, Jenkinson M, Johansen-Berg H, *et al.* Tract-based spatial statistics: Voxelwise analysis of multi-subject diffusion data. *Neuroimage* 2006; **31**: 1487–505.

14 Andersson JLR, Jenkinson M, Smith S. Non-linear registration, aka spatial normalisation. FMRIB Technial Report TR07JA2. *Oxford Cent Funct Magn Reson Imaging Brain, Dep Clin Neurol Oxford Univ Oxford, UK* 2007; : 22.

15 Andersson JLR, Jenkinson M, Smith SM. Non-linear optimisation. FMRIB technical report TR07JA1. *In Pract* 2007; : 16.

16 Mori S, Zijl P Van. Human white matter atlas. *Am J Psychiatry* 2007; **164**: 75390.

17 Hua K, Zhang J, Wakana S, *et al.* Tract Probability Maps in Stereotaxic Spaces: Analyses of White Matter Anatomy and Tract-Specific Quantification. *Neuroimage* 2008; **39**: 336–47.
